# Evidence That Higher Temperatures Are Associated With a Marginally Lower Incidence of COVID-19 Cases

**DOI:** 10.3389/fpubh.2020.00367

**Published:** 2020-07-10

**Authors:** Anne Meyer, Rohan Sadler, Céline Faverjon, Angus Robert Cameron, Melanie Bannister-Tyrrell

**Affiliations:** ^1^Ausvet Europe, Lyon, France; ^2^Ausvet, Fremantle, WA, Australia

**Keywords:** COVID-19, SARS-CoV-2, coronavirus, pandemic, temperature, humidity

## Abstract

Seasonal variations in COVID-19 incidence have been suggested as a potentially important factor in the future trajectory of the pandemic. Using global line-list data on COVID-19 cases reported until 17th of March 2020 and global gridded weather data, we assessed the effects of air temperature and relative humidity on the daily incidence of confirmed COVID-19 local cases at the subnational level (first-level administrative divisions). After adjusting for surveillance capacity and time since first imported case, average temperature had a statistically significant, negative association with COVID-19 incidence for temperatures of −15°C and above. However, temperature only explained a relatively modest amount of the total variation in COVID-19 cases. The effect of relative humidity was not statistically significant. These results suggest that warmer weather may modestly reduce the rate of spread of COVID-19, but anticipation of a substantial decline in transmission due to temperature alone with onset of summer in the northern hemisphere, or in tropical regions, is not warranted by these findings.

## Introduction

Pandemic COVID-19, caused by a beta-coronavirus named SARS-CoV-2 first identified in Wuhan, China (1), has spread rapidly. This spread was pronounced in temperate regions of the northern hemisphere, coinciding with winter (2). The number of cases reported in countries in tropical regions is lower (2), with most low- and middle-income countries having weaker detection and response capacity (3). To date, spread of COVID-19 has been minimal in high income southern hemisphere countries such as Australia and New Zealand, which were in their summer season when the first cases were reported at the end of January and February, respectively (4, 5). There has been much speculation about whether warmer temperatures are associated with decreased COVID-19 transmission, similar to what is observed for many viral respiratory infections (6). Higher temperatures were shown to have a protective effect against transmission of severe acute respiratory syndrome (SARS) in 2002–2003 (7), possibly due to the decreased survival of the SARS-CoV on surfaces at higher temperatures (8). Decreased aerosol spread at higher temperatures is another possible mechanism, as observed for human influenza viruses (9), though the role of aerosols in SARS-CoV-2 transmission remains unclear (10–13).

Several studies have investigated the association between weather variation (principally temperature and humidity) and COVID-19 spread (14–18). However, there are several important limitations of studies published to date. Firstly, existing studies have not distinguished between imported and locally acquired infections. This is potentially a significant source of bias in existing studies, as imported infections are not related to weather conditions at the location at which they are detected. For example, 62.5% of COVID-19 cases in Australia (as of May 10th 2020) were acquired overseas (19), and the proportion was even higher earlier in the pandemic. Secondly, most studies have not taken variation in capacity to detect emerging infections into account—this is particularly relevant for interpreting data on the spread of COVID-19 in the first few weeks of the pandemic. Finally, no studies have conducted a global analysis using COVID-19 data consistently aggregated at subnational level, which reflects limitations of current COVID-19 reporting. For example, a recent global analysis (17) had COVID-19 data available at a mixture of city, province and country level. Country-level COVID-19 data was matched to weather data for the capital city, which masks significant weather variation that can occur within countries.

At present, consistent global datasets on COVID-19 cases, or the public health interventions implemented in response to COVID-19, are not available at subnational level. This significantly limits efforts to disentangle effects of weather variation from effects of public health interventions since widespread “lockdown” and other substantial control measures were initiated. However, detailed COVID-19 data from the first few weeks of the pandemic, prior to widespread implementation of interventions following the declaration of a pandemic, could be informative for understanding the association between COVID-19 and weather variation. A partially complete global open line list of all COVID-19 cases reported since the start of the pandemic, including detailed location and epidemiological information for each case, presents an opportunity for detailed analysis of COVID-19 and weather at subnational level (20). Therefore, this study aimed to analyze seasonal variation in COVID-19 at subnational level, taking limitations of existing studies into account.

## Methods

### Study Design

This population-based open cohort study investigated the effect of weather-related variables (air temperature and relative humidity) on daily COVID-19 case counts at the beginning of the pandemic. The daily case count was modeled at the level of the first-level administrative division (ADM1) in which they occurred, by constructing a daily time series of COVID-19 cases based on the date of case confirmation for each ADM1.

### Setting and Participants

An open-source line list of confirmed COVID-19 cases was downloaded on March 18th 2020 (20). The line list included data on laboratory-confirmed cases from December 29th 2019 up to March 17th 2020 for all countries, including China. Cases included patients who had been admitted for treatment in hospitals and patients who did not require hospital admission. At that stage, the COVID-19 outbreak had just been declared a global pandemic by the World Health Organization (on March 11th 2020). Over 179,000 cases had been confirmed in 100 countries (21). Although community transmission was already confirmed in many countries of the Western Pacific and European regions, most countries only announced stringent national measures (“lockdowns”) the week following the pandemic declaration, or later.

All ADM1 associated with at least one confirmed case of COVID-19 in the source dataset were included in the analysis, excluding Hubei province in China. Within these ADM1, all cases for which either a date of case confirmation or a date of onset of symptoms was available were included in the analysis.

Hubei province was excluded from the analysis as case reports of unusual pneumonia-like illness precede confirmation of the first confirmed COVID-19 case in Hubei province by several weeks (i.e., the observation period is incomplete), and case confirmation was likely substantially delayed or missed for many early cases. Further, it remains unknown whether a single or multiple spillover event(s) initiated transmission in Hubei. Ongoing animal to human transmission alongside human to human transmission may have occurred early in the outbreak, and it is unclear what the impact of weather conditions would have been on these spillover events. Last, widespread implementation of interventions started substantially earlier in Hubei than in the rest of China and the world.

### Variables

#### Outcome Variable

We modeled the daily count of COVID-19 cases classified as local cases in each ADM1, from the date of first case report in the ADM1 to March 17th. Confirmed cases from the line list were classified as imported when travel history was reported in the associated data or as local otherwise.

#### Exposure Variables

We assumed that the weather variables would influence the transmission of SARS-CoV-2 at the time of infection. The dates of case confirmation were available, while the dates of infection were estimated as follows. The minimum time from infection to confirmation was estimated to be 3 days (22). The maximum time from infection to confirmation was estimated at 20 days, comprising an incubation period of up to 14 days (22) and the time to seek medical diagnosis and obtain a laboratory confirmation, which was estimated at up to 6 days (value estimated from the data). This value is close to the median of 7 days reported between the onset of symptoms and hospital admission reported in Wuhan (1). Therefore, the primary exposure variables were the mean air temperature and humidity at the ADM1 centroid between 3 and 20 days before the date of case confirmation. The temperature variable was included both as simple and squared terms to allow for non-linear associations with the outcome. Due to model convergence issues, the humidity variable was only included as a simple term.

#### Potential Confounders

Four variables were included in the model as potential confounders: the time since the first reported case in the ADM1 (to account for right-censoring), the median age of the national population (United Nations database, https://ourworldindata.org/age-structure, to account for the higher incidence of severe cases in older people, which may be more readily detected), the population density in the ADM1 (Socioeconomic Data and Application Center, https://sedac.ciesin.columbia.edu) and the capacity of the country to detect an emerging infectious disease. The Global Health Security Index (GHSI) (https://www.ghsindex.org/) publishes a country-level score (out of 100) for capacity for “early detection and reporting for epidemics of potential concern.” This indicator is a weighted average of indicators related to laboratory systems, real-time surveillance, and reporting, epidemiology workforce, and data integration between human, animal, and environmental health sectors.

### Data Sources

#### Spatial Data Sources and Processing

Spatial data on ADM1 were obtained from the Global Administrative Areas dataset (https://gadm.org/, accessed March 4th 2020). This corresponds to the first-level administrative unit within each country, usually described as a state or province. The reported coordinates of each confirmed case (variably a point location, city centroid, or different subnational administrative levels) were used to determine the ADM1 in which the case occurred.

#### Weather Data Sources and Processing

Daily gridded temperature data at 0.5-degree spatial resolution were obtained from the Climate Prediction Centre (NOAA/OAR/ESRL PSD, Boulder, Colorado, USA, https://www.esrl.noaa.gov/psd/data/gridded/data.cpc.globaltemp.html, accessed March 18th 2020). The daily temperature at the ADM1 centroid was calculated by taking the average of the maximum and minimum temperatures at the centroid coordinates for each day in the time series. Missing values for a given 0.5-degree cell and day were imputed from, by order of preference: the temperature in the neighboring spatial cells (Moore neighborhood) on the same day, the temperature for the previous or next day in the same cell, the relevant temperature from another dataset from the same source, the NCEP Daily Global Analyses. This dataset contains analyzed gridded temperature data at 2.5-degree spatial resolution (https://www.esrl.noaa.gov/psd/data/gridded/data.ncep.html, accessed March 18th 2020). Last, daily surface-level relative humidity data at 2.5-degree spatial resolution were obtained from the same dataset (NCEP Daily Global Analyses). The daily relative humidity at the ADM1 centroid was extracted for each day in the time series. The processing of weather data was performed in the R environment (23) using packages ncdf4 (24) and rgdal (25).

### Statistical Methods

The statistical model was based on the generalized linear mixed effect regression framework, using a negative binomial distribution, implemented through the glmmTMB package (26). A zero-inflation component, with no predictor variables, was added to account for the large proportion of zero observed in the daily time series. Continuous variables were centered and scaled. The ADM1 location was included as a random effect (27). Initial data exploration indicated the presence of autocorrelation in the model results, that was adequately controlled for by adding an autoregression term of order 2. Diagnostic plots as well as model validation tests were obtained using the DHARMa package (28), to assess the distribution of predicted values, the presence of outliers, as well as residual dispersion and zero inflation. The bias-adjusted Akaike information criterion (AICc) was used to compare related models: the null model (no fixed effects, random effect only), a full model with all the variables described above, and three nested models obtained by removing the temperature and humidity variables, one at a time and together. The dataset and *R* script used for statistical modeling are provided as Supplementary Materials.

## Results

**Figure 1:**
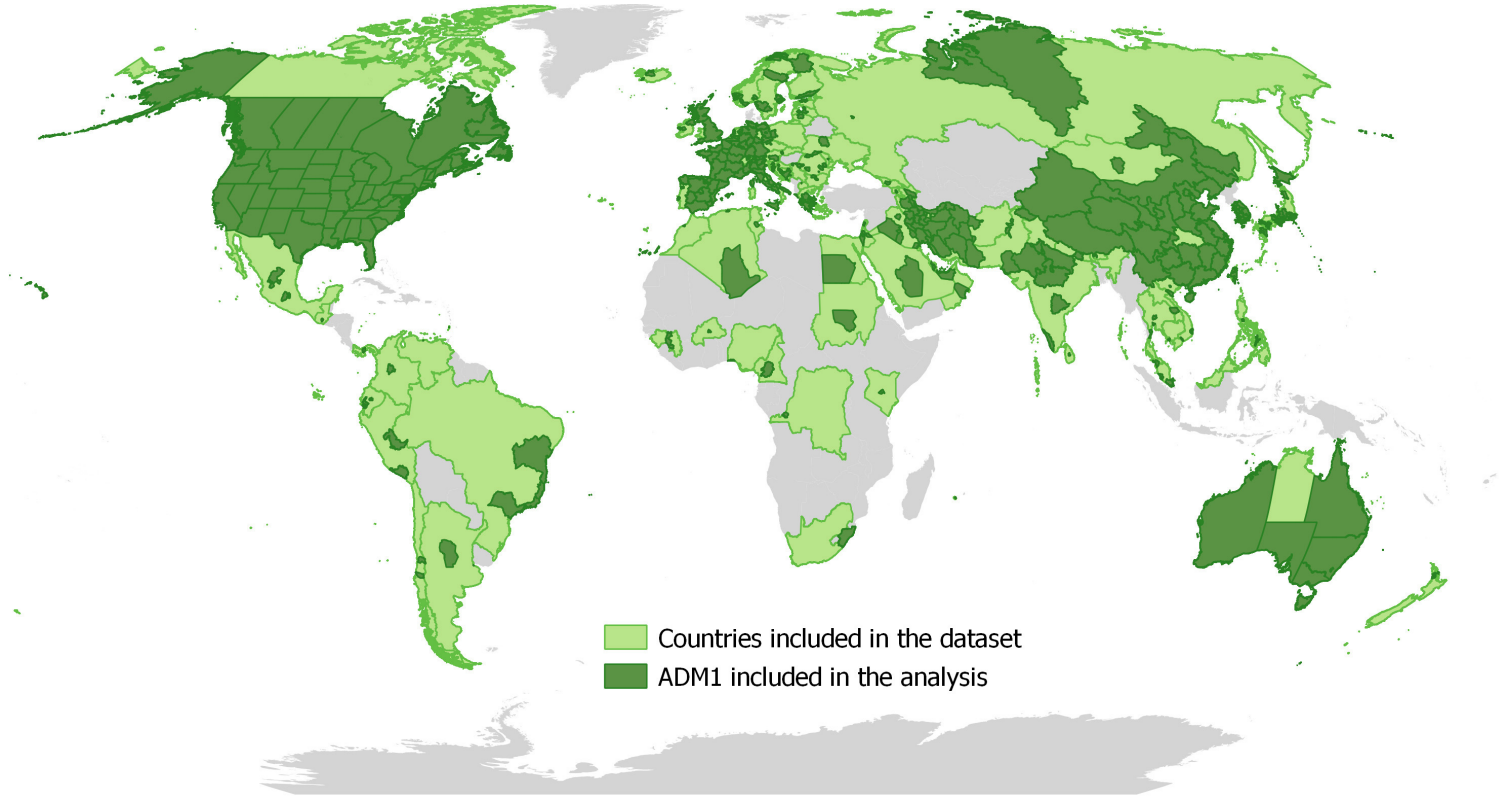
Distribution of ADM1 units that reported at least one COVID-19 case up to March 18th 2020.

As of March 18th 2020, the line list contained detailed data on 26,032 cases, from which 25,861 cases had a valid confirmation date entry and were used for the analysis. One additional case only had the date of onset of symptoms, and its case confirmation was estimated to have occurred 6 days later, based on the mean delay observed in the data. A total of 407 ADM1 units worldwide reported at least one case and were included in the model. This included 30 provinces in China as well as 377 ADM1-level reports in 99 other countries (Figure 1). There were 2,322 daily, ADM1-level observations with at least one reported case (Table 1).

**Table 1:** Summary statistics for the dataset used in a study of the effect of air temperature and humidity on the incidence of local COVID-19 cases (data as of March 18th 2020).

**Variable**	**Type of observation**	**Min**	**25th percentile**	**Median**	**Mean**	**75th percentile**	**Max**	**s.d.**
Daily number of imported cases	China (excl. Hubei)	0	0	0	1	1	19	2
	Outside China	0	0	0	1	1	32	2
Daily number of local cases	China (excl. Hubei)	0	1	6	16	19	258	26
	Outside China	0	0	1	9	5	485	26
Daily air temperature (°C)	China (excl. Hubei)	−26.4	−2.5	3.7	2.6	9.5	24.0	10.2
	Outside China	−33.9	4.5	8.6	9.7	14.2	34.3	9.6
Daily air humidity (%)	China (excl. Hubei)	22.3	56.7	71.8	69.4	84.2	98.8	17.6
	Outside China	6.0	55.0	70.8	67.1	80.7	99.5	18.0
Early detection GHSI score (country-level)	All	9	49	70	68	92	98	22

Model comparison showed that the full model and the model including the temperature variables and confounding variables only provided a similar fit to the data (Table 2). Excluding the relative humidity variable did not significantly modify the AICc. However, excluding the temperature variables led to a substantial increase in AICc. The marginal pseudo R-squared was 21% for the full model, decreasing to 13% after removing the temperature effect.

**Table 2:** Comparison of nested models of the incidence of local COVID-19 cases.

**Model formula**	**AICc**	**ΔAICc**	**Adjusted number of parameters**	**Marginal pseudo R-squared**	**Conditional pseudo R-squared**
Confounding variables + temperature + humidity	18,291	0	13	0.21	0.45
Confounding variables + temperature	18,293	2	12	0.20	0.44
Confounding variables only	18,371	80	10	0.13	0.38
Confounding variables + humidity	18,372	81	11	0.13	0.39
Null model	19,077	785	4	0.00	0.36

**Figure 2:**
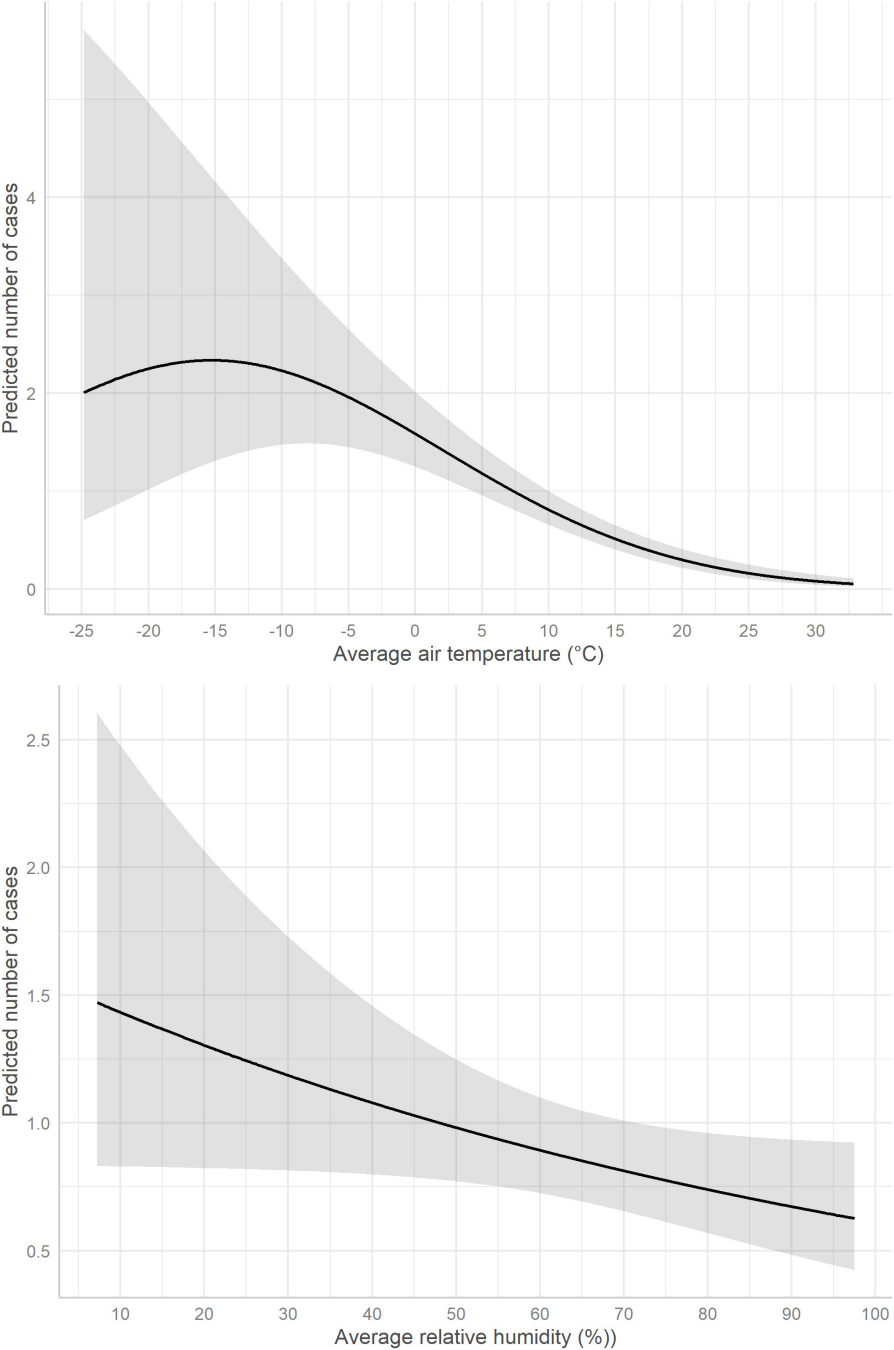
Predicted daily number of local cases of COVID-19 by 1st-level administrative unit according to average air temperature (upper panel) and relative humidity (lower panel) from 3 to 20 days before case confirmation. The gray area represents the 95% prediction interval.

The confounding variables corresponding to the population characteristics (median age and population density) were not significant predictors of the daily COVID-19 incidence (Table 3). The early detection capacity of the country had a statistically significant, positive association with the outcome. The time since the first case confirmation in the ADM1 had a statistically significant, negative association with the outcome. Air temperature has a statistically significant quadratic association with the case incidence: an increase in air temperature was associated with a decreasing incidence for temperatures above −15°C (Figure 2). The relative humidity had a negative association with the case incidence which was not statistically significant.

**Table 3:** Parameter estimates from the full model of incidence of local COVID-19 cases.

**Variable**	**Coefficient estimate and 95% confidence interval (log scale)**	***P*-value**
Intercept (zero-inflation model)	−0.76 [−0.94; −0.58]	<0.001
Intercept (conditional model)	−0.23 [−0.44; −0.02]	0.030
Time since first case	−0.63 [−0.71; −0.56]	<0.001
Temperature	−0.88 [−1.08; −0.67]	<0.001
Temperature, squared	−0.19 [−0.29; −0.08]	<0.001
Relative humidity	−0.14 [−0.29; 0.00]	0.052
Early detection score	0.30 [0.13; 0.46]	0.001
Population density	−0.10 [−0.32; 0.13]	0.397
Median population age	−0.07 [−0.27; 0.13]	0.512

*Coefficients are presented for the scaled variables*.

## Discussion

This study provides new evidence for the impact of weather-related parameters on the incidence of COVID-19 cases. There was a statistically significant effect of the average air temperature during the 3 preceding weeks on the COVID-19 case incidence in our study. However, the effect size was quite small, as shown by the pseudo R-squared estimates and changes in predicted values. The COVID-19 case incidence was negatively correlated with the air temperature for temperature above −15°C. Notably, the effect of relative humidity was not statistically significant. This study provides evidence that there may be seasonal variability in transmission of SARS-CoV-2, but this analysis does not imply that temperature alone is a primary driver of COVID-19 transmission. The observed association may not be due directly to temperature, but to correlated factors such as human behaviors during cold weather.

Countries with higher early detection capacity had a higher reported case incidence. We suggest that this association is due to a detection bias, where countries with better disease detection capacity simply detect more cases. Current reports of the pandemic show that almost all countries across the globe have been affected by SARS-CoV-2, despite the large variance in their capacity to prevent, detect, and respond to disease outbreaks (29). However, we expected the opposite association, where countries with higher early detection capacity would have lower cases due to their ability to implement control measures earlier. Surprisingly, the association of the time since the first case confirmation in the ADM1 with the outcome was negative. We believe this is linked to considerable underreporting of cases in the global data source used for this study. Manual assessment of the time series showed that as the time since the first case increased, the number of cases reported for each ADM1 did not follow the expected exponential pattern. We suggest that this is due to the overwhelming number of cases confirmed as the outbreak becomes more severe, resulting in limited availability of information on individual cases after the initial stages. The two issues discussed here are common in epidemiological analyses based on reported cases.

These results complement those of several recently published studies investigating the weather effect published for China (14–16), Brazil (30), Spain (31), and at a global level (17, 18). There are also many related studies not yet peer-reviewed and available as pre-prints. The published studies for China and Brazil as well as one of the global studies showed a negative association between the air temperature and COVID-19 case or mortality incidence, using different lag periods (14–16, 18, 30). The two other studies did not find evidence of a relationship between COVID-19 cases and air temperature (17, 31). Four studies showed a negative association between relative or absolute humidity and COVID-19 incidence (14, 16–18) while a fifth showed that an increase in relative humidity was associated with an increase in number of COVID-19 cases (15).

There are several strengths to this analysis, which add to the evidence base for an association between weather variation and COVID-19. Most importantly, this study made use of detailed line list data, which enabled the first global analysis of COVID-19 cases at province or state level, and for the categorization of COVID-19 cases as local or imported. The relevance of this potential bias is evident when considering countries such as Australia, where over 60% of COVID-19 cases to date were acquired overseas. Nonetheless, there are several important limitations to our analysis. The line list data used for this analysis were incomplete, compared to globally reported cases. Furthermore, despite using detailed case data, there was no consistent data available on many characteristics that affect the rate of spread within a region, especially the interventions initiated in response to the detection of imported or locally transmitted cases. Including data on implemented interventions to contain or mitigate COVID-19 in further analysis would provide additional insights into the effect of weather-related parameters.

Temperature and humidity have also been considered as factors influencing the spread of pandemic influenza and other respiratory tract viruses. Human pandemic influenza tends to show few seasonal trends upon emergence, while seasonal patterns appear during subsequent waves (32). These patterns have been linked with a more efficient transmission in cold and dry weather, in particular via aerosols (9, 33). However, numerous other factors linked to the host, virus and environment are likely to play a role (34). Aerosol experiments on the 2009 H1N1 virus showed that the virus had a similar sensitivity to temperature and humidity as known seasonal influenza viruses (35). The authors suggested that the unusual timing of the H1N1 pandemic, with a high incidence in summer and autumn, may have been due to the lack of population immunity, which played a larger role in disease spread than temperature and humidity related factors. Our results regarding the effect of temperature on COVID-19 incidence are consistent with some of these characteristics. The possibility of similar recurrence and seasonality has been suggested for SARS-CoV-2 (36), though caution is warranted before extrapolating characteristics observed for pandemic influenza to pandemic COVID-19.

## Conclusion

This study provides evidence of a modest association between warmer temperatures and lower COVID-19 incidence, for cases reported globally until March 17th 2020. Therefore, warmer weather may modestly reduce the rate of spread of COVID-19, but anticipation of a substantial decline in transmission due to temperature alone with onset of summer in the northern hemisphere, or in tropical regions, is not warranted by these findings.

## Data Availability Statement

Publicly available datasets were analyzed in this study. This data can be found here: https://tinyurl.com/s6gsq5y (COVID-19 data) and https://www.esrl.noaa.gov/psd/data/gridded (weather data).

## Ethics Statement

The analysis made use of publicly available, anonymised data only. Therefore, no approval to conduct the study from an Ethical Review Board was sought. Nonetheless, the study was conducted in accordance with the Declaration of Helsinki, as revised in 2013.

## Author Contributions

AM, AC, CF, and MB-T contributed to the conception and design of the study. AM, AC, and CF processed the data. AM and RS performed the statistical analysis. AM and MB-T wrote the first draft of the manuscript. All authors contributed to the manuscript revision and read and approved the submitted version.

## Conflict of Interest

All authors are employed by the Ausvet Group via its companies Ausvet and Ausvet Europe.

## References

[1] Huang C Wang Y Li X Ren L Zhao J Hu Y. Clinical features of patients infected with 2019 novel coronavirus in Wuhan, China. Lancet. (2020) 395:497–506. 10.1016/S0140-6736(20)30183-5 31986264 PMC7159299

[2] World Health Organization Coronavirus Disease 2019 (COVID-19) Situation Report−111. Geneva (2020) Available online at: https://www.who.int/docs/default-source/coronaviruse/situation-reports/20200510covid-19-sitrep-111.pdf?sfvrsn=1896976f_2 (accessed 12 March, 2020).

[3] Gilbert M Pullano G Pinotti F Valdano E Poletto C Boëlle P-Y. Preparedness and vulnerability of African countries against importations of COVID-19: a modelling study. Lancet. (2020) 395:871–7. 10.1016/S0140-6736(20)30411-6 32087820 PMC7159277

[4] World Health Organization Coronavirus Disease 2019 (COVID-19) Situation Report−5. Geneva (2020). Available online at: https://www.who.int/docs/default-source/coronaviruse/situation-reports/20200125-sitrep-5-2019-ncov.pdf?sfvrsn=429b143d_8 (accessed March 12, 2020).

[5] World Health Organization Coronavirus Disease 2019 (COVID-19) Situation Report−39. Geneva (2020). Available online at: https://www.who.int/docs/default-source/coronaviruse/situation-reports/20200228-sitrep-39-covid-19.pdf?sfvrsn=5bbf3e7d_4 (accessed March 12, 2020).

[6] Johnson HC Gossner CM Colzani E Kinsman J Alexakis L Beauté J. Potential scenarios for the progression of a COVID-19 epidemic in the European Union and the European Economic Area, March 2020. Eurosurveillance. (2020) 25:2000202. 10.2807/1560-7917.ES.2020.25.9.2000202 32156332 PMC7068161

[7] Lin K Fong DY-T Zhu B Karlberg J. Environmental factors on the SARS epidemic: air temperature, passage of time and multiplicative effect of hospital infection. Epidemiol Infect. (2006) 134:223–30. 10.1017/S0950268805005054 16490124 PMC2870397

[8] Chan KH Peiris JSM Lam SY Poon LLM Yuen KY Seto WH. The effects of temperature and relative humidity on the viability of the SARS coronavirus. Adv Virol. (2011) 2011:734690. 10.1155/2011/734690 22312351 PMC3265313

[9] Lowen AC Mubareka S Steel J Palese P. Influenza virus transmission is dependent on relative humidity and temperature. PLOS Pathog. (2007) 3:e151. 10.1371/journal.ppat.0030151 17953482 PMC2034399

[10] Morawska L Cao J. Airborne transmission of SARS-CoV-2: the world should face the reality. Environ Int. (2020) 139:105730. 10.1016/j.envint.2020.105730 32294574 PMC7151430

[11] van Doremalen N Bushmaker T Morris DH Holbrook MG Gamble A Williamson BN. Aerosol and surface stability of SARS-CoV-2 as compared with SARS-CoV-1. N Engl J Med. (2020) 382:1564–7. 10.1056/NEJMc2004973 32182409 PMC7121658

[12] Cheng VCC Wong S-C Chen JHK Yip CCY Chuang VWM Tsang OTY. Escalating infection control response to the rapidly evolving epidemiology of the coronavirus disease 2019 (COVID-19) due to SARS-CoV-2 in Hong Kong. Infect Control Hosp Epidemiol. (2020) 41:493–8. 10.1017/ice.2020.58 32131908 PMC7137535

[13] Ong SWX Tan YK Chia PY Lee TH Ng OT Wong MSY. Air, surface environmental, and personal protective equipment contamination by severe acute respiratory syndrome coronavirus 2 (SARS-CoV-2) from a symptomatic patient. JAMA. (2020) 323:1610–2. 10.1001/jama.2020.3227 32129805 PMC7057172

[14] Ma Y Zhao Y Liu J He X Wang B Fu S. Effects of temperature variation and humidity on the death of COVID-19 in Wuhan, China. Sci Total Environ. (2020) 724:138226. 10.1016/j.scitotenv.2020.138226 32408453 PMC7142681

[15] Pirouz B Shaffiee Haghshenas S Shaffiee Haghshenas S Piro P Investigating a serious challenge in the sustainable development process: analysis of confirmed cases of COVID-19 (new type of coronavirus) through a binary classification using artificial intelligence and regression analysis. Sustainability. (2020) 12:2427 10.3390/su12062427

[16] Qi H Xiao S Shi R Ward MP Chen Y Tu W. COVID-19 transmission in Mainland China is associated with temperature and humidity: a time-series analysis. Sci Total Environ. (2020) 728:138778. 10.1016/j.scitotenv.2020.138778 32335405 PMC7167225

[17] Jüni P Rothenbühler M Bobos P Thorpe KE da Costa BR Fisman DN. Impact of climate and public health interventions on the COVID-19 pandemic: a prospective cohort study. CMAJ. (2020) 192:566–73. 10.1503/cmaj.200920 32385067 PMC7259972

[18] Wu Y Jing W Liu J Ma Q Yuan J Wang Y. Effects of temperature and humidity on the daily new cases and new deaths of COVID-19 in 166 countries. Sci Total Environ. (2020) 729:139051. 10.1016/j.scitotenv.2020.139051 32361460 PMC7187824

[19] Department of Health. Australian COVID-19 Cases by Source of Infection. Australian Government–Department of Health. (2020). Available online at: https://www.health.gov.au/resources/australian-covid-19-cases-by-source-of-infection (accessed November 5, 2020).

[20] Xu B Kraemer MUG Xu B Gutierrez B Mekaru S Sewalk K. Open access epidemiological data from the COVID-19 outbreak. Lancet Infect Dis. (2020) 20:534. 10.1016/S1473-3099(20)30119-5 32087115 PMC7158984

[21] World Health Organization Coronavirus Disease 2019 (COVID-19) Situation Report−57. Geneva (2020). Available online at: https://www.who.int/docs/default-source/coronaviruse/situation-reports/20200317-sitrep-57-covid-19.pdf?sfvrsn=a26922f2_4 (accessed 12 March, 2020).

[22] Lauer SA Grantz KH Bi Q Jones FK Zheng Q Meredith HR. The incubation period of coronavirus disease 2019 (COVID-19) from publicly reported confirmed cases: estimation and application. Ann Intern Med. (2020) 10:M20–0504. 10.7326/M20-0504 32150748 PMC7081172

[23] R Core Team R: A Language and Environment for Statistical Computing. Vienna (2019). Available online at: https://www.R-project.org/ (accessed May 12, 2020).

[24] Pierce D ncdf4: Interface to Unidata netCDF (Version 4 or Earlier) Format Data Files. R package version 1.17. (2019). Available online at: https://CRAN.R-project.org/package=ncdf4 (accessed May 12, 2020).

[25] Bivand R Keitt T Rowlingson B rgdal: Bindings for the “Geospatial” Data Abstraction Library. R Package Version 1.4-8. (2019). Available online at: https://CRAN.R-project.org/package=rgdal (accessed May 12, 2020).

[26] Brooks M Kristensen K van Bethem K Magnusson A Berg C Nielsen A glmmTMB balances speed and flexibility among packages for zero-inflated generalized linear mixed modeling. R J. (2017) 9:378–400. 10.32614/RJ-2017-066

[27] Wood SN Stable and efficient multiple smoothing parameter estimation for generalized additive models. J Am Stat Assoc. (2004) 99:673–86. 10.1198/016214504000000980

[28] Hartig F DHARMa: Residual Diagnostics for Hierarchical (Multi-Level/Mixed) Regression Models. (2019). Available online at: https://CRAN.R-project.org/package=DHARMa (accessed May 12, 2020).

[29] Kandel N Chungong S Omaar A Xing J. Health security capacities in the context of COVID-19 outbreak: an analysis of International Health Regulations annual report data from 182 countries. Lancet Lond Engl. (2020) 395:1047–53. 10.1016/S0140-6736(20)30553-5 32199075 PMC7271261

[30] Prata DN Rodrigues W Bermejo PH. Temperature significantly changes COVID-19 transmission in (sub)tropical cities of Brazil. Sci Total Environ. (2020) 729:138862. 10.1016/j.scitotenv.2020.138862 32361443 PMC7182516

[31] Briz-Redón Á Serrano-Aroca Á. A spatio-temporal analysis for exploring the effect of temperature on COVID-19 early evolution in Spain. Sci Total Environ. (2020) 728:138811. 10.1016/j.scitotenv.2020.138811 32361118 PMC7194829

[32] Kilbourne ED. Influenza pandemics of the 20th century. Emerg Infect Dis. (2006) 12:9–14. 10.3201/eid1201.051254 16494710 PMC3291411

[33] Lowen AC Steel J Mubareka S Palese P. High temperature (30°C) blocks aerosol but not contact transmission of influenza virus. J. Virol. (2008) 82:5650–2. 10.1128/JVI.00325-08 18367530 PMC2395183

[34] Moriyama M Hugentobler WJ Iwasaki A. Seasonality of respiratory viral infections. Ann Rev Virol. (2020) 7:1. 10.1146/annurev-virology-012420-022445 32196426

[35] Steel J Palese P Lowen AC. Transmission of a 2009 Pandemic influenza virus shows a sensitivity to temperature and humidity similar to that of an H3N2 seasonal strain. J Virol. (2011) 85:1400–02. 10.1128/JVI.02186-10 21084485 PMC3020521

[36] Kissler SM Tedijanto C Goldstein E Grad YH Lipsitch M. Projecting the transmission dynamics of SARS-CoV-2 through the postpandemic period. Science. (2020) 368:860–8. 10.1126/science.abb5793 32291278 PMC7164482

[37] Bannister-Tyrrell M Meyer A Faverjon C Cameron A Preliminary evidence that higher temperatures are associated with lower incidence of COVID-19, for cases reported globally up to 29th February 2020. medRxiv. (2020). 10.1101/2020.03.18.20036731 PMC7365860 32754568

