# Supplementary material for: Evidence That Higher Temperatures Are Associated With a Marginally Lower Incidence of COVID-19 Cases
Source: Front Public Health. 2020 Jul 10;8:367. doi: 10.3389/fpubh.2020.00367 (PMC7365860; doi:10.3389/fpubh.2020.00367)
Supplement: Supplementary file 2 [file Data_Sheet_2.PDF]

```
# Description:   Data processing and analysis for the paper "Evidence that higher temperatures are associated with a marginally lower incidence of COVID-19 cases"

# Date:          2020-06-12

# Note:          all nc files were obtained from https://www.esrl.noaa.gov/psd/data/gridded


# ---------------------------

# Load libraries

library(dplyr)

library(tidyr)

library(ncdf4)

library(sp)

library(rgdal)

library(glmmTMB)

library(ggplot2)

library(ggeffects)


# ---------------------------

# Load dataset (case list pre-processed in PostgreSQL to add geographic information and additional variables)

myData <- read.csv(file = "data/case_list_20200318.csv", na.strings=c("","NA"))


# ---------------------------

# 1. Match climate data to case data


allData <- myData  %>%

  group_by(adm1_longitude_360 = ifelse(adm1_longitude>=0,adm1_longitude,360+adm1_longitude)) %>%

  mutate(date_case = as.Date(date_case,"%Y-%m-%d"))%>%

  as.data.frame()


myLocations <- unique(allData[,c("adm1_gid","adm1_longitude","adm1_latitude","adm1_longitude_360")])

myLocationsGid <- unique(allData$adm1_gid)

allData <- subset(allData, is.na(date_case)==FALSE)

myDates <- seq(min(allData$date_case)-28,max(allData$date_case),1)

climateDataset <- expand.grid(adm1_gid=myLocationsGid,date_case=myDates)
```

```
climateDataset <- climateDataset %>%
  mutate(date_case_hours=as.numeric(difftime(date_case, as.Date("1900-01-01"), units = "hours"))) %>%
  mutate(date_case_days=as.numeric(difftime(date_case, as.Date("1800-01-01"), units = "days"))) %>%
  as.data.frame()

climateDataset <- merge(climateDataset,myLocations,by="adm1_gid",all.X=T)

# ---------------------------
# 1.1 Air temperatures from 2.5° grid
varname <- "air"

# 2020
filename <- "data/air.sfc.2020.nc"
ncin <- nc_open(filename)
tmp <- ncvar_get(ncin,varname)
lon <- ncvar_get(ncin, "lon")
lat <- ncvar_get(ncin, "lat")
time <- ncvar_get(ncin, "time")
climateDataset$temperature_avg<-NA

for (i in 1:nrow(climateDataset)) {
longitude_index<-which(abs(lon-climateDataset$adm1_longitude_360[i])==min(abs(lon-
climateDataset$adm1_longitude_360[i])))[1]
latitude_index<-which(abs(lat-climateDataset$adm1_latitude[i])==min(abs(lat-climateDataset$adm1_latitude[i])))[1]
if (climateDataset$date_case_days[i]>=80353){
time_index<-which(time == climateDataset$date_case_days[i])
climateDataset$temperature_avg[i] <- tmp[longitude_index,latitude_index,time_index] - 273.15 # convert from
Kelvin to Celsius
}}

nc_close(ncin)
rm(ncin)
gc()

# 2019
```

```
filename <- "data/air.sfc.2019.nc"
ncin <- nc_open(filename)
tmp <- ncvar_get(ncin,varname)
lon <- ncvar_get(ncin, "lon")
lat <- ncvar_get(ncin, "lat")
time <- ncvar_get(ncin, "time")

for (i in 1:nrow(climateDataset)) {
longitude_index<-which(abs(lon-climateDataset$adm1_longitude_360[i])==min(abs(lon-
climateDataset$adm1_longitude_360[i])))[1]
latitude_index<-which(abs(lat-climateDataset$adm1_latitude[i])==min(abs(lat-climateDataset$adm1_latitude[i])))[1]
if (climateDataset$date_case_days[i]<80353){
time_index<-which(time == climateDataset$date_case_days[i])
climateDataset$temperature_avg[i] <- tmp[longitude_index,latitude_index,time_index] - 273.15
}
}

nc_close(ncin)
rm(ncin,tmp,lon,lat,time)
gc()

# 1.2. Humidity from 2.5° grid
varname <- "rhum"

# 2020
filename <- "data/rhum.sfc.2020.nc"
ncin <- nc_open(filename)
tmp <- ncvar_get(ncin,varname)
lon <- ncvar_get(ncin, "lon")
lat <- ncvar_get(ncin, "lat")
time <- ncvar_get(ncin, "time")
climateDataset$humidity<-NA

for (i in 1:nrow(climateDataset)) {
```

```
longitude_index<-which(abs(lon-climateDataset$adm1_longitude_360[i])==min(abs(lon-climateDataset$adm1_longitude_360[i])))[1]

latitude_index<-which(abs(lat-climateDataset$adm1_latitude[i])==min(abs(lat-climateDataset$adm1_latitude[i])))[1]

if (climateDataset$date_case_days[i]>=80353){

time_index<-which(time == climateDataset$date_case_days[i])

climateDataset$humidity[i] <- tmp[longitude_index,latitude_index,time_index]

}}


nc_close(ncin)

rm(ncin,tmp,lon,lat,time)

gc()


# 2019

filename <- "data/rhum.sfc.2019.nc"

ncin <- nc_open(filename)

tmp <- ncvar_get(ncin,varname)

lon <- ncvar_get(ncin, "lon")

lat <- ncvar_get(ncin, "lat")

time <- ncvar_get(ncin, "time")


for (i in 1:nrow(climateDataset)) {

longitude_index<-which(abs(lon-climateDataset$adm1_longitude_360[i])==min(abs(lon-climateDataset$adm1_longitude_360[i])))[1]

latitude_index<-which(abs(lat-climateDataset$adm1_latitude[i])==min(abs(lat-climateDataset$adm1_latitude[i])))[1]

if (climateDataset$date_case_days[i]<80353){

time_index<-which(time == climateDataset$date_case_days[i])

climateDataset$humidity[i] <- tmp[longitude_index,latitude_index,time_index]

}

}


nc_close(ncin)

rm(ncin,tmp,lon,lat,time)

gc()


# 1.3.  Minimum air temperatures from 0.5° grid (where available)
```

```
varname <- "tmin"


# 2020
filename <- "data/tmin.2020-03-30.nc"
ncin <- nc_open(filename)
tmp <- ncvar_get(ncin,varname)
lon <- ncvar_get(ncin, "lon")
lat <- ncvar_get(ncin, "lat")
time <- ncvar_get(ncin, "time")
climateDataset$temperature_min<-NA


for (i in 1:nrow(climateDataset)) {
longitude_index<-which(abs(lon-climateDataset$adm1_longitude_360[i])==min(abs(lon-
climateDataset$adm1_longitude_360[i])))[1]
latitude_index<-which(abs(lat-climateDataset$adm1_latitude[i])==min(abs(lat-climateDataset$adm1_latitude[i])))[1]
if (climateDataset$date_case_hours[i]>=1051896){
time_index<-which(time %in% (seq(climateDataset$date_case_hours[i],climateDataset$date_case_hours[i]+23,1)))
# if missing value, get value from neighbour cell in space or time
climateDataset$temperature_min[i] <- ifelse(is.na(mean(tmp[longitude_index,latitude_index,time_index]))==FALSE,
   mean(tmp[longitude_index,latitude_index,time_index]),
   mean(tmp[(max(1,longitude_index-1)):(min(length(lon),longitude_index+1)),(max(1,latitude_index-
1)):(min(length(lat),latitude_index+1)),max(1,(time_index-1)):min(length(time),(time_index+1))], na.rm=T)
)
}
}


nc_close(ncin)
rm(ncin,tmp,lon,lat,time)
gc()


# 2019
filename <- "data/tmin.2019.nc"
ncin <- nc_open(filename)
tmp <- ncvar_get(ncin,varname)
lon <- ncvar_get(ncin, "lon")
```

```
lat <- ncvar_get(ncin, "lat")

time <- ncvar_get(ncin, "time")


for (i in 1:nrow(climateDataset)) {

longitude_index<-which(abs(lon-climateDataset$adm1_longitude_360[i])==min(abs(lon-climateDataset$adm1_longitude_360[i])))[1]

latitude_index<-which(abs(lat-climateDataset$adm1_latitude[i])==min(abs(lat-climateDataset$adm1_latitude[i])))[1]

if (climateDataset$date_case_hours[i]<1051896){

time_index<-which(time %in% (seq(climateDataset$date_case_hours[i],climateDataset$date_case_hours[i]+23,1)))

climateDataset$temperature_min[i] <- ifelse(is.na(mean(tmp[longitude_index,latitude_index,time_index]))==FALSE,

   mean(tmp[longitude_index,latitude_index,time_index]),

   mean(tmp[(max(1,longitude_index-1)):(min(length(lon),longitude_index+1)),(max(1,latitude_index-1)):(min(length(lat),latitude_index+1)),max(1,(time_index-1)):min(length(time),(time_index+1))], na.rm=T)

)

}

}


nc_close(ncin)

rm(ncin,tmp,lon,lat,time)

gc()


# 1.4.  Maximum air temperatures from 0.5° grid (where available)

varname <- "tmax"


# 2020

filename <- "data/tmax.2020-03-30.nc"

ncin <- nc_open(filename)

tmp <- ncvar_get(ncin,varname)

lon <- ncvar_get(ncin, "lon")

lat <- ncvar_get(ncin, "lat")

time <- ncvar_get(ncin, "time")

climateDataset$temperature_max<-NA


for (i in 1:nrow(climateDataset)) {
```

```
longitude_index<-which(abs(lon-climateDataset$adm1_longitude_360[i])==min(abs(lon-climateDataset$adm1_longitude_360[i])))[1]

latitude_index<-which(abs(lat-climateDataset$adm1_latitude[i])==min(abs(lat-climateDataset$adm1_latitude[i])))[1]

if (climateDataset$date_case_hours[i]>=1051896){

time_index<-which(time %in% (seq(climateDataset$date_case_hours[i],climateDataset$date_case_hours[i]+23,1)))

climateDataset$temperature_max[i] <- ifelse(is.na(mean(tmp[longitude_index,latitude_index,time_index]))==FALSE,

   mean(tmp[longitude_index,latitude_index,time_index]),

   mean(tmp[(max(1,longitude_index-1)):(min(length(lon),longitude_index+1)),(max(1,latitude_index-1)):(min(length(lat),latitude_index+1)),max(1,(time_index-1)):min(length(time),(time_index+1))], na.rm=T)

)

}}


nc_close(ncin)

rm(ncin,tmp,lon,lat,time)

gc()


# 2019

filename <- "data/tmax.2019.nc"

ncin <- nc_open(filename)

tmp <- ncvar_get(ncin,varname)

lon <- ncvar_get(ncin, "lon")

lat <- ncvar_get(ncin, "lat")

time <- ncvar_get(ncin, "time")


for (i in 1:nrow(climateDataset)) {

longitude_index<-which(abs(lon-climateDataset$adm1_longitude_360[i])==min(abs(lon-climateDataset$adm1_longitude_360[i])))[1]

latitude_index<-which(abs(lat-climateDataset$adm1_latitude[i])==min(abs(lat-climateDataset$adm1_latitude[i])))[1]

if (climateDataset$date_case_hours[i]<1051896){

time_index<-which(time %in% (seq(climateDataset$date_case_hours[i],climateDataset$date_case_hours[i]+23,1)))

climateDataset$temperature_max[i] <- ifelse(is.na(mean(tmp[longitude_index,latitude_index,time_index]))==FALSE,

   mean(tmp[longitude_index,latitude_index,time_index]),

   mean(tmp[(max(1,longitude_index-1)):(min(length(lon),longitude_index+1)),(max(1,latitude_index-1)):(min(length(lat),latitude_index+1)),max(1,(time_index-1)):min(length(time),(time_index+1))], na.rm=T)

)

}}
```

```
nc_close(ncin)
rm(ncin,tmp,lon,lat,time)
gc()

# Prepare dataset
# Use higher precision temperature where available, lower precision otherwise
climateDataset$temperature<-
ifelse(is.na((climateDataset$temperature_max+climateDataset$temperature_min)/2)==TRUE,
                climateDataset$temperature_avg,
                (climateDataset$temperature_max+climateDataset$temperature_min)/2
                )
climateDataset$temperature_max<-NULL
climateDataset$temperature_min<-NULL
climateDataset$temperature_avg<-NULL
climateDataset$adm1_longitude_360<-NULL
climateDataset$date_case_hours<-NULL
climateDataset$date_case_days<-NULL
climateDataset$day<-climateDataset$date_case
climateDataset$date_case<-NULL

# --------------------------
# 2. Preparing data for modelling

endWindow<-20
startWindow<-3

# Drop cases with no date of symptoms or report (N=170)
myData<-droplevels(subset(myData,is.na(date_case)==FALSE))

myData$date_onset_symptoms<-NULL
myData$date_confirmation<-NULL
myData$is_imported<-ifelse(as.character(myData$is_imported)=="t",TRUE,FALSE)
```

```
# Aggregate data by day and ADM1

aggData <- myData %>%

  group_by(country, adm1_gid, adm1_latitude, adm1_longitude, date_case, early_score, median_age, pop_density)
%>%

  summarize(imported_cases=sum(is_imported), local_cases=sum(1-is_imported)) %>%

  as.data.frame()


aggData$adm1_gid<-as.factor(aggData$adm1_gid)

aggData$date_case<-as.Date(aggData$date_case,"%Y-%m-%d")


# Merge with climate data

names(climateDataset)[which(names(climateDataset)=="day")]<-"date_case"

climateDataset$date_case<-as.Date(as.character(climateDataset$date_case))

climateDataset$adm1_gid<-factor(climateDataset$adm1_gid,levels=levels(aggData$adm1_gid))


adm1Unique<-unique(aggData$adm1_gid)

dates <-  as.Date(min(climateDataset$date_case):max(climateDataset$date_case),origin="1970-01-01")

valuesAdm1<-rep(adm1Unique,each=length(dates))

dates <- rep(dates,length(adm1Unique))


empty<- data.frame(adm1_gid=valuesAdm1,date_case=dates)

emptyWhich<-which(names(aggData) %in% c("date_case","imported_cases","local_cases"))

empty<-inner_join(x=empty,y=aggData[,-emptyWhich],by=c("adm1_gid"))

empty<-unique(empty)

nonEmpty<- match(paste(empty$adm1_gid, empty$date_case),

                paste(aggData$adm1_gid, aggData$date_case))

nonEmpty<-which(!is.na(nonEmpty))

empty<-empty[-nonEmpty,]


empty$imported_cases<-0

empty$local_cases<-0

empty<-empty[,match(names(aggData),names(empty))]


combined <- rbind(aggData, empty)
```

```
combined <- combined %>%
  group_by(adm1_gid,date_case,.drop=TRUE)

combined<-with(combined,combined[order(adm1_gid,date_case),])
combined<-as.data.frame(combined)

combined<-
merge(x=combined,y=climateDataset[,c("humidity","temperature","adm1_gid","date_case")],by=c("adm1_gid","dat
e_case"))
combined<-combined[order(combined$adm1_gid,combined$date_case),]

combined$total_cases<-combined$imported_cases + combined$local_cases

combined <- combined %>%
  group_by(adm1_gid) %>%
  mutate(first_case=min(date_case[total_cases>0])) %>%
  as.data.frame()

combined <- combined %>%
  group_by(adm1_gid) %>%
  mutate(time_case=as.numeric(date_case-first_case)) %>%
  as.data.frame()

earlyOmitIndicator<-which(combined$time_case<0)

# Temperature and humidity on 20 days prior to case
tempMatrix<- matrix(NA,nrow=nrow(combined),ncol=endWindow)
humidityMatrix<- matrix(NA,nrow=nrow(combined),ncol=endWindow)
caseMatrix<- matrix(NA,nrow=nrow(combined),ncol=endWindow)

for (i in 1:length(adm1Unique)) {
  admWhich<-which(combined$adm1_gid==adm1Unique[i])
  for (j in admWhich) {
    for (k in 1:endWindow) {
```

```
      if ((j-k)>0) {
        if (combined$adm1_gid[(j-k)]==adm1Unique[i]) {
    tempMatrix[j,k]<- combined$temperature[(j-k)]
    humidityMatrix[j,k]<- combined$humidity[(j-k)]
    caseMatrix[j,k]<- combined$total_cases[(j-k)]
      }}
    }
  }
}

allData<-combined[-earlyOmitIndicator,]
tempMatrix<-tempMatrix[-earlyOmitIndicator,]
humidityMatrix<-humidityMatrix[-earlyOmitIndicator,]
caseMatrix<- caseMatrix[-earlyOmitIndicator,]

# Extract temperature and humidity mean for each case during time frame of interest
tempMean<-apply(tempMatrix[,startWindow:endWindow],1,sum)/ncol(tempMatrix[,startWindow:endWindow])
humidityMean<-
apply(humidityMatrix[,startWindow:endWindow],1,sum)/ncol(humidityMatrix[,startWindow:endWindow])

# Scale continuous variables
allData$early_scoreSC<-(allData$early_score-mean(allData$early_score))/sd(allData$early_score)
allData$pop_densitySC<-(allData$pop_density-mean(allData$pop_density))/sd(allData$pop_density)
allData$median_ageSC<-(allData$median_age-mean(allData$median_age))/sd(allData$median_age)
allData$time_caseSC<-(allData$time_case-mean(allData$time_case))/sd(allData$time_case)
tempMeanSC<-(tempMean-mean(tempMean))/sd(tempMean)
humidityMeanSC<-(humidityMean-mean(humidityMean))/sd(humidityMean)

# Autocorrelation terms for case time series
caseLag1<-caseMatrix[,1]
caseLag2<-caseMatrix[,2]

# ---------------------------
# 3. Statistical modelling
```

```
modelFull <- glmmTMB(local_cases~
  time_caseSC +
  tempMeanSC +
  I(tempMeanSC^2) +
  humidityMeanSC +
  early_scoreSC +
  pop_densitySC +
  median_ageSC +
  (1|adm1_gid) +
  caseLag1 +
  caseLag2,
data=allData,
ziformula=~1,
family=nbinom1
)

modelnoT <- glmmTMB(local_cases~
  time_caseSC +
#  tempMeanSC +
#  I(tempMeanSC^2) +
  humidityMeanSC +
  early_scoreSC +
  pop_densitySC +
  median_ageSC +
  (1|adm1_gid) +
  caseLag1 +
  caseLag2,
data=allData,
ziformula=~1,
family=nbinom1
)

modelnoH <- glmmTMB(local_cases~
```

```
  time_caseSC +
  tempMeanSC +
  I(tempMeanSC^2) +
#  humidityMeanSC +
  early_scoreSC +
  pop_densitySC +
  median_ageSC +
  (1|adm1_gid) +
  caseLag1 +
  caseLag2,
data=allData,
ziformula=~1,
family=nbinom1
)

modelnoTH <- glmmTMB(local_cases~
  time_caseSC +
#  tempMeanSC +
#  I(tempMeanSC^2) +
#  humidityMeanSC +
  early_scoreSC +
  pop_densitySC +
  median_ageSC +
  (1|adm1_gid) +
  caseLag1 +
  caseLag2,
data=allData,
ziformula=~1,
family=nbinom1
)

modelNull <- glmmTMB(local_cases~ 1+
#  tempMeanSC +
#  I(tempMeanSC^2) +
```

```
#  humidityMeanSC +
#  early_scoreSC +
#  pop_densitySC +
#  median_ageSC +
  (1|adm1_gid),
#  caseLag1 +
#  caseLag2,
data=allData,
ziformula=~1,
family=nbinom1
)

# ---------------------------
# 4. Model outputs

tempE<-ggpredict(modelFull, "tempMeanSC [all]", type = "fe")
myLabels<-seq(-25,30,5)
myBreaks<-(myLabels-mean(tempMean))/sd(tempMean)
plot1<-plot(tempE) +
labs(x = "Average air temperature (°C)", y = "Predicted number of cases") +
theme(plot.title = element_blank()) +
scale_x_continuous(breaks=myBreaks, labels=myLabels)

humE<-ggpredict(modelFull, "humidityMeanSC [all]", type = "fe")
myLabels<-seq(0,100,10)
myBreaks<-(myLabels-mean(humidityMean))/sd(humidityMean)
plot2<-plot(humE) +
labs(x = "Average relative humidity (%))", y = "Predicted number of cases") +
theme(plot.title = element_blank()) +
scale_x_continuous(breaks=myBreaks, labels=myLabels)

tiff(file = "figure2.tif", width = 2000, height = 3000, res=300)
gridExtra::grid.arrange(plot1, plot2, ncol=1, nrow = 2)
dev.off()
```

```
MuMIn::model.sel(list(modelFull,modelnoH,modelnoTH,modelnoT,modelNull))


performance::r2(modelFull)

performance::r2(modelnoH)

performance::r2(modelnoTH)

performance::r2(modelnoT)

performance::r2(modelNull)


myCI<-confint(modelFull)[c(1,2,3,4,5,6,7,8,9,10,12),]

myCoef<-rbind(summary(modelFull)$coefficients$cond,summary(modelFull)$coefficients$zi)

modelCI<-cbind(myCI,myCoef)

modelCI<-as.data.frame(modelCI)
```
